# Cathepsin S and Protease-Activated Receptor-2 Drive Alloimmunity and Immune Regulation in Kidney Allograft Rejection

**DOI:** 10.3389/fcell.2020.00398

**Published:** 2020-06-05

**Authors:** Yutian Lei, Benjamin Ehle, Santhosh V. Kumar, Susanne Müller, Solange Moll, Andrew F. Malone, Benjamin D. Humphreys, Joachim Andrassy, Hans-Joachim Anders

**Affiliations:** ^1^Division of Nephrology, Department of Medicine IV, University Hospital, LMU Munich, Munich, Germany; ^2^Division for General, Visceral, Transplant, Vascular and Thoracic Surgery, University Hospital, LMU Munich, Munich, Germany; ^3^Department of Pathology, University of Munich, Munich, Germany; ^4^Institute of Clinical Pathology, University Hospital Geneva, Geneva, Switzerland; ^5^Division of Nephrology, Department of Medicine, Washington University in Saint Louis School of Medicine, St. Louis, MO, United States; ^6^Department of Developmental Biology, Washington University in Saint Louis School of Medicine, St. Louis, MO, United States

**Keywords:** kidney transplantation, cathepsin S, allorejection, proteinase-activated receptor-2, animal model of transplantation

## Abstract

Alloantigen presentation is an essential process in acute allorejection. In this context, we speculated on a pathogenic role of cathepsin S (Cat-S), a cysteine protease known to promote antigenic peptide loading into MHC class II and to activate protease-activated receptor (PAR)-2 on intrarenal microvascular endothelial and tubular epithelial cells. Single-cell RNA sequencing and immunostaining of human kidney allografts confirmed Cat-S expression in intrarenal mononuclear phagocytes. *In vitro*, Cat-S inhibition suppressed CD4 + T cell lymphocyte activation in a mixed lymphocyte assay. *In vivo*, we employed a mouse model of kidney transplantation that showed preemptive Cat-S inhibition significantly protected allografts from tubulitis and intimal arteritis. To determine the contribution of PAR-2 activation, first, Balb/c donor kidneys were transplanted into Balb/c recipient mice without signs of rejection at day 10. In contrast, kidneys from C57BL/6J donor mice revealed severe intimal arteritis, tubulitis, interstitial inflammation, and glomerulitis. Kidneys from *Par2*-deficient C57BL/6J mice revealed partial protection from tubulitis and lower intrarenal expression levels for *Fasl*, *Tnfa*, *Ccl5*, and *Ccr5*. Together, we conclude that Cat-S and PAR-2 contribute to immune dysregulation and kidney allograft rejection, possibly involving Cat-S-mediated activation of PAR-2 on recipient parenchymal cells in the allograft.

## Introduction

Among the different forms of renal replacement, therapy kidney transplantation, when available, is the preferred option for most patients with end-stage kidney disease (Wolfe et al., 1999; Robinson et al., 2016). Alloimmunity remains an important factor limiting graft survival, and life-long treatment with relatively non-specific immunosuppressants has remained the standard of care to date for the vast majority of patients (Durrbach et al., 2010). More selective interference with alloimmunity may broaden the range of options for those patients facing drug toxicity.

Presentation of alloantigen’s is a central path mechanism of alloimmunity and involves MHC class II molecules on professional antigen-presenting cells (Borges et al., 2016; Robson et al., 2018). Within antigen-presenting cells, the maturation of MHC class II molecules is tightly regulated, whereby the invariant chain covers the peptide-binding domain up to when peptide loading occurs and the molecule is shuttled to the cell surface (Roche and Frusta, 2015; Robson et al., 2018). Cathepsin (Cat-) S is one of several proteases that chop the invariant chain in a stepwise process (Shi et al., 1999; Roche and Frusta, 2015); hence, Cat-S deficiency or pharmaceutical Cat-S inhibition prevents MHC class II–mediated (auto)antigen presentation (Rise et al., 1996, 1998; Dresden et al., 1999; Saugus et al., 2002; Stickle et al., 2012). Indeed, Cat-S inhibition effectively suppresses the immune dysregulation in numerous experimental autoimmune diseases (Saugus et al., 2002; Baugh et al., 2011; Rupanagudi et al., 2015; Tato et al., 2017).

Beyond its role inside cells, monocytes/macrophages, neutrophils, and endothelial cells secrete Cat-S in the extracellular space where it processes several matrix proteins (Lutgens et al., 2007; Reiser et al., 2010). For example, its elastase activity contributes to vascular wall degeneration in atherosclerosis and aortic aneurysm formation (Shi et al., 2003; Sukhova et al., 2003; Rodgers et al., 2006; Aikawa et al., 2009; Samokhin et al., 2010; Qin et al., 2012; Figueiredo et al., 2015). Indeed, increased serum levels of Cat-S are associated with several cardiovascular risk factors including chronic kidney disease (Jobs et al., 2011, 2013; Lv et al., 2012). As a new finding, we and others recently described that Cat-S activates protease-activated receptor (PAR)-2 on vascular endothelial cells in a thrombin-like manner (Elmariah et al., 2014; Zhao et al., 2014; Kumar et al., 2016). Cat-S-driven PAR-2 activation induced endothelial dysfunction, a central path mechanism in microvascular complications of diabetic mellitus or systemic autoimmunity (Kumar et al., 2016; Tato et al., 2017).

As both mechanisms, MHC class II–mediated antigen presentation and microvascular injury also contribute to immune dysregulation and allograft dysfunction in solid organ transplantation, we hypothesized that interfering with either Cat-S or PAR-2 would attenuate organ injury in a robust model of allograft rejection. To address this concept, we decided for a rigorous mouse model of acute kidney allograft rejection without further immunosuppressive therapy.

## Materials and Methods

### Single-Cell RNA Sequencing of Human Kidney Biopsies

#### Tissue Processing

The renal biopsy was minced into small pieces with a razor blade and incubated at 37°C in freshly prepared dissociation buffer containing 0.25% trypsin and 40 U/ml DNase I, filtered resuspended in buffer (9% OptiPrep). Nuclei from normal human nephrectomy tissue were isolated with Nuclei EZ Lysis buffer with protease inhibitor and RNase inhibitor. Samples were cut into <2-mm pieces, homogenized, and incubated on ice for 5 min with an additional 2 ml of lysis buffer. The homogenate was filtered, centrifuged, resuspended in suspension Buffer (1 × PBS, 0.07% BSA, 0.1% RNase inhibitor), and counted.

#### InDrops Single-Cell RNA-Seq

InDrops was performed as described (Klein et al., 2015). In brief, cells were diluted into 60,000 cells/ml in 9% OptiPrep buffer. Single-cell encapsulation was carried out using an inDrops instrument and microfluidic chip manufactured by 1CellBio. In total, 4000 cells were collected. Library preparation was performed according to the protocol provided by the manufacturer. Libraries were sequenced by HiSeq 2500 with a sequencing depth of 50K mapped reads/cell.

#### 10× Single-Nucleus RNA-Seq

RNAs from 6000 single nuclei loaded into one lane of the 10 × Genomics 3-prime V2 platform were encapsulated, barcoded, and reversed transcribed. The library was sequenced in HiSeq 2500 with a sequencing depth of 12.5K mapped reads/cell.

#### Data Preprocessing

We used the inDrops computational pipeline, dropEst (Klein et al., 2015), to process the single-cell InDrops data. We used STAR to map the high-quality reads to the human genome (GRCh38). We next ran the dropEst program to estimate the accurate molecular counts, which generated a UMI count matrix for each gene in each cell. We used the zUMIs computational pipeline to process the single nuclei data according to protocol (Parekh et al., 2018). In brief, fastq files were filtered for low-quality barcodes and unique molecular identifier (UMIs). Next, cDNA reads were mapped to the reference genome using STAR. Count matrices were generated for exon + intron overlapping reads. These count matrices were used for downstream analysis.

#### Unsupervised Clustering and Cell Type Identification

UMI count matrices were loaded into the R package Seurat. For normalization, the DGE matrix was scaled by total UMI counts, multiplied by 10,000, and transformed to log space. Only genes found to be expressed in >10 cells were retained. Cells with a relatively high percentage of UMIs mapped to mitochondrial genes (≥0.3) were discarded. Moreover, cells with fewer than 300 or more than 4,000 detected genes were omitted, resulting in 4,487 cells. We also regressed out the variants arising from library size and percentage of mitochondrial genes using the function RegressOut in R package Seurat. The highly variable genes were identified using the function MeanVarPlot with the parameters x.low.cutoff = 0.0125, x.high.cutoff = 6, and y.cutoff = 1, resulting in an output of 2,404 highly variable genes. The expression level of highly variable genes in the cells was scaled and centered along each gene and was conducted to principal component analysis. Based on PCElbowPlot and PCHeatmap Seurat function analysis, we first selected 20 PCs for two-dimensional t-distributed stochastic neighbor embedding (tSNE), implemented by the Seurat software with the default parameters. Based on the tSNE map, sixteen clusters were identified using the function FindCluster in Seurat with the resolution parameter set to 0.6. We applied the same unsupervised clustering analysis on the single nucleus dataset. After filtering low-quality nuclei, 4,609 nuclei with >400 genes expressed were imported into Seurat for clustering analysis. In total, we identified 13 cell types in the single nucleus dataset, which included macrophages and endothelial cells. Integrated analysis of rejecting and normal human kidney was performed using the Seurat function IntegrateData.

### Immunohistochemistry in Human Renal Biopsies

Human renal tissue, fixed in formaldehyde and embedded in paraffin, was selected from the files of the Service of Pathology, University Hospital Geneva: control normal renal tissue was obtained from two patients with nephrectomy performed for neoplasia, involving the possibility of tumor-related immune exhaustion. Five biopsy specimens were obtained from renal transplant patients with acute T cell–mediated rejection. For all biopsy specimens, standard analyses were performed. Each patient gave informed consent before enrollment. The institutional ethical committee board approved the clinical protocol (CEREH number 03-081). The research was performed according to the Helsinki’s declaration principles. For immunostaining, serial paraffin sections were stained with the primary antibodies anti-cathepsin S (monoclonal mouse anti-human Cat-S, LSBio, Seattle, WA, United States) and anti-CD68 (DakoCytomation, Glostrup, Denmark) or double stained with anti-cathepsin S and anti-CD68. Counterstaining was performed using Mayer hematoxylin. Negative controls included the absence of the primary antibody (not shown).

### Bone Marrow–Derived Dendritic Cell Isolation and Differentiation

For bone marrow–derived dendritic cell (BMDC) preparation, cells were isolated and cultured according to a standard method with minor modifications (Han et al., 1999). Briefly, bone marrow cells were cultured in RPMI 1640 media supplemented with 1% penicillin and streptomycin, 10% of fetal calf serum (S0115, EMD Millipore, United States), 20 ng/ml of mouse recombinant IL-4 and GM-CSF (ImmunoTools, Friesoythe, Germany). At day 8, non-adherent cells were transferred to a fresh plate, primed by 500 μg/ml lipopolysaccharide (LPS, Sigma) for another 24 h, and used for MLR. In BMDC stimulation assays, non-adherent cells were transferred to 12-well plate at 2 × 10^6^/ml at day 8, stimulated with indicated stimuli for 24 h, and analyzed by flow cytometry.

### Mixed Lymphocyte Reaction

For *in vitro* assessment of allogenic T-cell activation, mixed lymphocyte reaction (MLR) was set up by incubating T cell–enriched splenocytes together with bone marrow–derived dendritic cells (BMDCs). C57BL/6 (H-2b) and Balb/c (H-2d) mice were used at the age of 7–15 weeks. For T cell preparation, pan T-cells were enriched from splenocytes by a magnetic bead–based negative selection method (Mouse Pan T-cell Isolation Kit II, Miltenyi Biotec, Germany) according to the manufacturer’s instruction (purity >90%, data not shown). Purified T cells were labeled with 5 μM carboxyfluorescein succinimidyl ester (CFSE) dye (CellTrace^TM^ CFSE Cell Proliferation Kit, Invitrogen) for 5 min according to the manufacturer’s instruction. For proliferation assay, 1.5 × 10^5^ CFSE-labeled T cells and 1 × 10^5^ of primed BMDCs were cocultured in round bottom 96-well plate (Nunc, Germany) for 4 days. Mixed cells were afterward analyzed by flow cytometry to evaluate the proliferation.

### Flow Cytometry Analysis

Single-cell suspensions from BMDC stimulation assay or MLR were washed in cold DPBS (PAN Biotech, Germany) twice and suspended in cold FACS buffer (DPBS with 1% BSA and 0.05% sodium azide). Single-cell suspensions were first treated with anti-mouse CD16/32 antibody (BioLegend, United States). Cells from BMDC stimulation assay were stained for anti-mouse CD11c-PE (clone HL3, BioLegend, United States) and anti-mouse MHCII-FITC (clone M5/114.15.2, BioLegend, United States). Cells from MLR were stained for anti-mouse CD8-PE (clone 53-6,7, BioLegend, United States) and then stained for anti-mouse CD4-APC antibody (RM4-4 clone, BioLegend, United States). Samples were analyzed on a flow cytometry analyzer (BD FACSCalibur). For analysis of proliferation, after gating in live/CD4 + CD8- or live/CD4-CD8 +, CFSE histograms were deconvoluted to differentiate each daughter generation from parent cells by software (FlowJo, version 7.6.5) (Supplementary Figure S1A). Division index was calculated by the ratio of the total number of divisions over the number of cells at start of culture.

### Lactate Dehydrogenase Cytotoxicity Assay

Lactate dehydrogenase (LDH) cytotoxicity assay was set up by mixing 1.5 × 10^5^ of CFSE-stained T cells and 1 × 10^5^ of LPS-primed BMDCs in RPMI 1640 media supplemented with 1% penicillin and streptomycin and 10% of fetal calf serum. Cells were incubated for 4 days. At day 4, cell death was evaluated using LDH cell cytotoxicity assay kit (Roche, Mannheim, Germany) according to the manufacturer’s protocol.

### Animal Study Design

C57BL/6J (H2b) and Balb/c (H2d) mice were obtained from Charles River (Sulzfeld, Germany) and used at the age of 8–12 weeks. *Par2*-/- mice in the C57BL/6J background were purchased from Jackson Laboratory (Bar Harbor, ME, United States). Offspring were genotyped by polymerase chain reaction (PCR) of genomic DNA derived from tail clippings. Animals were assigned by stratified randomization to different groups co-housed in groups of five in filter top cages with unlimited access to food and water. Cages, nestlets, food, and water were sterilized by autoclaving before use. Humane endpoints were monitored throughout the study. All experiments were conducted according to the European equivalent of the NIH’s Guide for the Care and Use of Laboratory Animals and had been approved by the local government authorities.

Kidney transplantations were performed as previously described (Skoskiewicz et al., 1973). Following a midline abdominal incision, the left kidney, aorta, and inferior vena cava of the donor were fully exposed and mobilized. The kidney was procured en bloc including the renal vein; the renal artery, along with a small aortic cuff; and the ureter. The vessels of the graft were anastomosed end-to-side to the recipient’s abdominal aorta and inferior vena cava using 10-0 nylon sutures (AROSurgical, Newport Beach, CA, United States). For urinary tract reconstruction, the ureter was directly anastomosed into the bladder using a pull-through (Han et al., 1999). The times of cold and warm ischemia of the graft were maintained at 40 and 30 min, respectively. The native kidneys of the recipient remained untouched as this was a non-life-sustaining approach.

**Primary Endpoint:** Harvested allografts were split in half and either paraffin embedded or snap frozen and kept at −80°C. Light microscopy was performed on HE- and PAS-stained whole cross sections of kidney allografts. An experienced blinded nephropathologist (S. M.) evaluated and scored for tubulitis, intimal arteritis, interstitial inflammation, and glomerulitis as well as periarteritis using a 4-point-score (0–3) and assigned a score according to the Banff criteria (Haas et al., 2014).

**Secondary Endpoints:** Real-time reverse transcription-polymerase chain reaction (RT-PCR). Gene expression was assessed by real-time quantitative RT-PCR as described (Lech et al., 2010). In brief, total RNA was isolated using an RNA extraction kit (Life Technologies, Darmstadt, Germany) according to the manufacturer’s instructions. After isolation of RNA, cDNA was generated using reverse transcriptase (Superscript II; Invitrogen, Carlsbad, CA, United States). A SYBR Green Dye detection system was used for quantitative real-time PCR on Light Cycler 480 (Roche, Mannheim, Germany) using SYBR Green (SABiosciences) as marker and 18s rRNA as a housekeeping gene. Gene-specific primers blasted with ensemble-BLAST and NCBI primer-BLAST (Metabion, Martinsried, Germany) were used. The following are forward and reverse gene-specific primers, respectively (300 nM; Metabion, Martinsried, Germany): 18s, GCAATTATTCCCCATGAACG and AGG GCCTCACTAAACCATCC; *Ifng*, TGAGCTCATTGAATGCTT GG and ACAGCAAGGCGAAAAAGGAT; *Fasl*, TTAAATGGG CCACACTCCTC and ACTCCGTGAGTTCACCAACC; *Ctss*, GAGTCCCATAGCCAACCACAAG and AAGCGGTGTCTATG ACGACCC; and *Ccl5*, GTGCCCACGTCAAGGAGTAT and CCACTTCTTCTCTGGGTTGG; *Ccr5*, GTCTACTTTCTCTT CTGGACTCC and CCAAGAGTCTCTGTTGCCTGCA; *Foxp3*, CTGGACACCCATTCCAGACT and TTCATGCATCAGCTC TCCAC; *Il2ra*, GCGTTGCTTAGGAAACTCCTGG and GCATA GACTGTGTTGGCTTCTGC, *Cd8b1*, GAATGTGAAGCCAGA GGACAGTG and GGGCAGTTGTAGGAAGGACATC; *Cd4*, GTTCAGGACAGCGACTTCTGGA and GAAGGAGAACTCC GCTGACTCT. Non-template controls consisting of all used reagents were negative for target and housekeeping genes. To reduce the risk of false-positive crossing point, the high-confidence algorithm was used. The melting curve profiles were analyzed for every sample to detect eventual unspecific products or primer dimers.

Histology was a secondary endpoint. Kidneys were fixed in 4% formalin, embedded in paraffin. Immunostaining was performed as described using anti-mouse MHC-II (1:100, clone M5/114.15.2, eBioscience, United States) (Kumar et al., 2016).

### Statistical Analysis

Normal data distribution was tested using the Shapiro–Wilk test. Comparisons between two groups were performed with Student’s *t*-test or Mann–Whitney *U* test. Comparison of multiple groups was performed with ANOVA or Kruskal–Wallis test; a multiple comparison test was performed with Dunnett or Dunn’s correction, respectively. A value of *p* < 0.05 was considered to indicate statistical significance. Data are presented as mean ± SD.

## Results

### Cathepsin S–Positive Cells Accumulate in Rejecting Human Kidney Allografts

**FIGURE 1:**
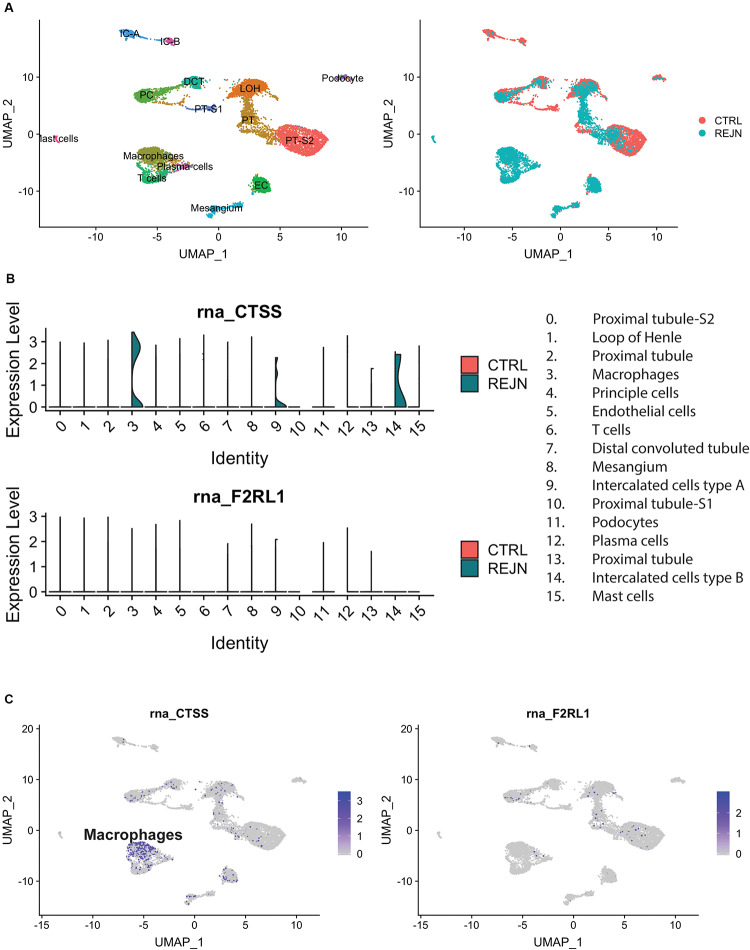
Cat-S and PAR-2 gene expression in healthy and rejected human kidney biopsies by single-cell RNA-seq. **(A)** UMAP plots of combined correlation analysis of a mixed rejection kidney transplant biopsy and a healthy human kidney tissue sample. Left: cell clusters labeled by cell type. Right: clusters labeled according to rejection (blue) or healthy (red) kidney. **(B)** Violin plots of Cat-S (*CTSS*) and PAR-2 (*F2RL1*) gene expression per cell type, in rejection (green) and healthy (red). Upper: *CTSS* expression. Lower: *F2RL1* expression. **(C)** Feature UMAP plots of Cat-S (left) and PAR2 (right) expression. Purple color demotes high gene expression.

**FIGURE 2:**
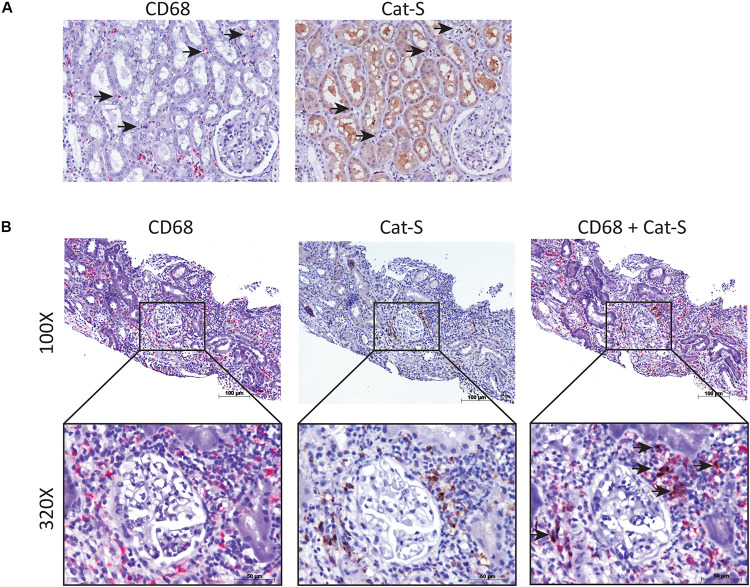
Cat-S expression in kidney biopsies from renal transplant patients with acute T cell–mediated rejection by immunostaining. **(A)** Cat-S and CD68 staining in health human kidney tissue. Consecutive kidney slides were stained for CD68 (left, in red) and Cat-S (right, in brown). CD68-positive cells and Cat-S-positive cells were marked by black arrows. Magnification 200×. **(B)** Cat-S and CD68 staining in rejecting human kidney allografts. CD68 single staining (left), Cat-S single staining (middle), and Cat-S/CD68 double staining (right) was performed on tissues from patients undergoing kidney rejection. Cat-S/CD68 double-positive cells were marked by black arrow.

We compared single-cell Cat-S expression (CTSS) in human kidney allograft with mixed rejection and normal human kidney (Wu et al., 2018). Integrated analysis of rejecting and normal human kidney identified 16-cell clusters including all major tubular and immune cell types and endothelial cells (Figure 1A). Compared to normal kidney, high expression of CTSS is seen in macrophages and intercalated cells (Figures 1B upper, 1C left). To confirm these data, we performed immunostaining in biopsies from transplanted patients diagnosed with kidney allograft rejection, as well as biopsies from healthy controls. As shown in Figure 2A, Cat-S-positive cells were sparse in the interstitium of healthy kidneys and were most likely expressed by CD68 + cells. In contrast, we found numerous CD68/Cat-S double-positive cells accumulating in rejected allografts (Figure 2B). Together, Cat-S was strongly expressed inside human kidney allografts.

### Cathepsin S Inhibition Suppresses Alloimmune Lymphocyte Proliferation *in vitro*

**FIGURE 3:**
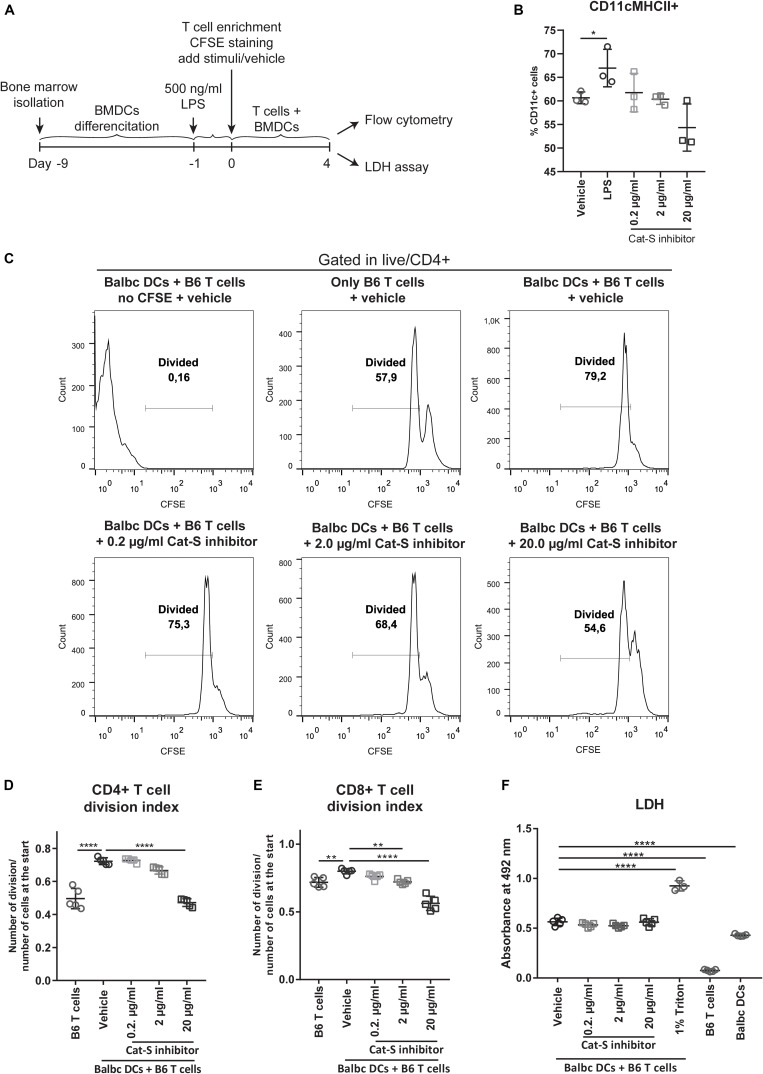
Effect of Cat-S inhibitor on MLR and cytotoxicity. **(A)** Experiment design of mixed lymphocyte reaction for proliferation and LDH assay. LPS-primed BMDCs (Balb/c) and CFSE-stained T cells (C57BL/6J) were used as stimulator and responder, respectively. Mixed cells were cocultured for 4 days and analyzed by flow cytometry or LDH assay. **(B)** BMDC stimulation assays. BMDCs were stimulated with 500 ng/ml LPS, Cat-S inhibitor, or vehicle for 24 h and analyzed for MHC-II expression among CD11c + cells. **(C)** CD4 + T cell proliferation monitored using CFSE labeling. Mixed cells were in the presence of 0.2, 2, 20 μg/ml, or vehicle, and proliferation was analyzed by flow cytometry at 4 days of culture. As controls, mixed cells with no CFSE were treated as the same way as vehicle but without CFSE staining; single T cells were treated with vehicle but without adding BMDCs. After gating in CD4 + cells, divided cells were gated in the histograms of the CFSE channel. A representative experiment from two separate experiments is shown. For each experiment, one mouse was used for BMDC differentiation and two mice were used for T cell isolation. Two or three replications was made from each T cell host for MLR. **(D)** Division index of CD4 + T cells. Based on the deconvoluted histograms of the CFSE channel, the division index was calculated by the ratio of the total number of divisions over the number of cells at start of culture. **(E)** The division index of CD8 + T cells. **(F)** LDH assay for Cat-s inhibitor–treated cells. Supernatant of mixed cell culture was analyzed at day 4 by LDH. *p < 0.05, **p < 0.01, ****p < 0.0001.

As Cat-S is a non-redundant component in MHC class II–driven antigen presentation, it should also drive MHC class II–related alloantigen presentation and alloimmunity. We tested this concept by performing mixed lymphocyte assays (Figure 3A) and measured CD4 + T lymphocyte division as a readout for alloantigen-specific lymphocyte activation. As shown in Figure 3B, Cat-S inhibition did not significantly affect BMDC MHC class II expression. In MLR, however, Cat-S inhibitor–treated groups had less proportion of divided CD4 + T cells (Figure 3C). Quantification of the division index also showed that the Cat-S inhibitor suppressed CD4 + T cell and CD8 + T cell division in a dose-dependent manner (Figures 3D,E). This effect was independent of cytotoxicity of the Cat-S inhibitor as LDH release was identical in all groups (Figure 3F). Thus, we conclude that Cat-S inhibition blocks alloimmune CD4 + and CD8 + T lymphocyte proliferation *in vitro*.

### Preemptive Cathepsin S Inhibition Attenuates Acute Kidney Allograft Rejection

**FIGURE 4:**
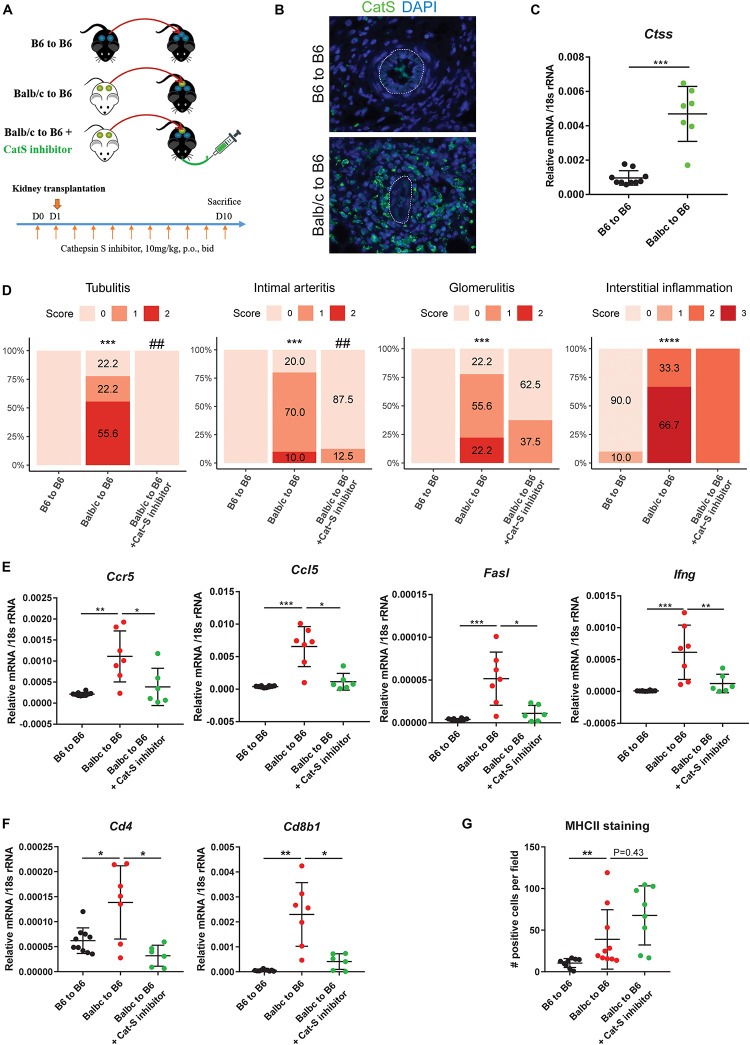
Cathepsin S inhibition attenuated renal allograft rejection *in vivo*. **(A)** Experimental design. Wild-type C57BL/6 kidneys (syngeneic) or wild-type Balb/c kidneys (allogeneic) were transplanted into wild-type C57BL/6 mice. Allogenic recipients were orally administrated with either vehicle or Cat-S inhibitor 10 mg/kg twice daily for a total of 11 days. At day 10 after transplantation, mice were analyzed. **(B)** Cat-S staining in mouse kidney grafts from syngeneic (B6 to B6) and allogeneic group (Balb/c to B6). White dash lines represent for vessels. Magnification 200×. **(C)** *Ctss* mRNA expression in mouse kidney grafts. **(D)** Histological score for mouse kidney grafts. Tubulitis, intimal arteritis, glomerulitis, and interstitial inflammation were quantified by Banff scoring method. ***p < 0.001 Balb/c to B6 vs. B6 to B6; ****p < 0.0001 Balb/c to B6 vs. B6 to B6; ^##^*p* < 0.01 Balb/c to B6 vs. Balb/c to B6 + Cat-S inhibitor. *N* = 10 for B6 to B6, *n* = 9–10 for Balb/c to B6, *n* = 8–10 for Balb/c to B6 + Cat-S inhibitor. **(E)** Proinflammation gene expression in mouse kidney grafts by RT-qPCR. **(F)** Cd4 and Cd8b1 gene expression in mouse kidney grafts. **(G)** Quantification of interstitial MHC-II positive cells in mouse kidney graft. *p < 0.05; **p < 0.01; ***p < 0.001. B6 represents for C56BL/6J.

Based on these findings, we tested whether Cat-S inhibition can attenuate kidney allograft rejection *in vivo*. We set up an experimental model based on C57BL/6J (H2b)–recipient mice (Figure 4A). We investigated the Cat-S expression in transplanted kidneys. Compared to syngeneic controls, allograft showed numerous Cat-S-positive cells accumulating in the interstitium, especially around vessels (Figure 4B). Likewise, Cat-S gene expression was also significantly induced in allograft (Figure 4C). Transplanting a kidney from a donor of the same strain showed minimal differences in the histological picture compared to the native kidneys 10 days after the surgery, implying that the transplant procedure *per se* was reliably performed and did not cause tissue damage by ischemia–reperfusion injury (Figure 4D). In contrast, kidneys from Balb/c (H2d) donor mice showed signs of severe rejection with tubulitis, glomerulitis, intimal arteritis, and interstitial inflammation (Figure 4D). Treating recipient mice with the Cat-S inhibitor RO5461111 completely abrogated tubulitis, intimal arteritis, but not glomerulitis and interstitial inflammation (Figure 4D). As a further sign of intrarenal inflammation, quantitative RT-PCR of total kidney RNA revealed increased levels of proinflammatory mediators such as *Ccl5*, *Ccr5*, *Fasl*, and *Ifng* (Figure 4E). Cat-S inhibition reduced intrarenal expression levels of above genes. Cat-S inhibition also reduced *Cd4* and *Cd8b1* gene expression (Figure 4F). However, it did not affect Foxp3 or Il2ra gene expression (Supplementary Figure S1B). The Cat-S inhibitor also did not affect MHC class II expression in graft (Figure 4G and Supplementary Figure S1D). Cat-S inhibition reduced intrarenal expression levels of the above genes (Figure 4E). Taken together, Cat-S inhibition substantially attenuates kidney allograft rejection.

### Lack of *Par2* in the Kidney Allograft Attenuates Acute Rejection in Recipient Balb/c Mice

**FIGURE 5:**
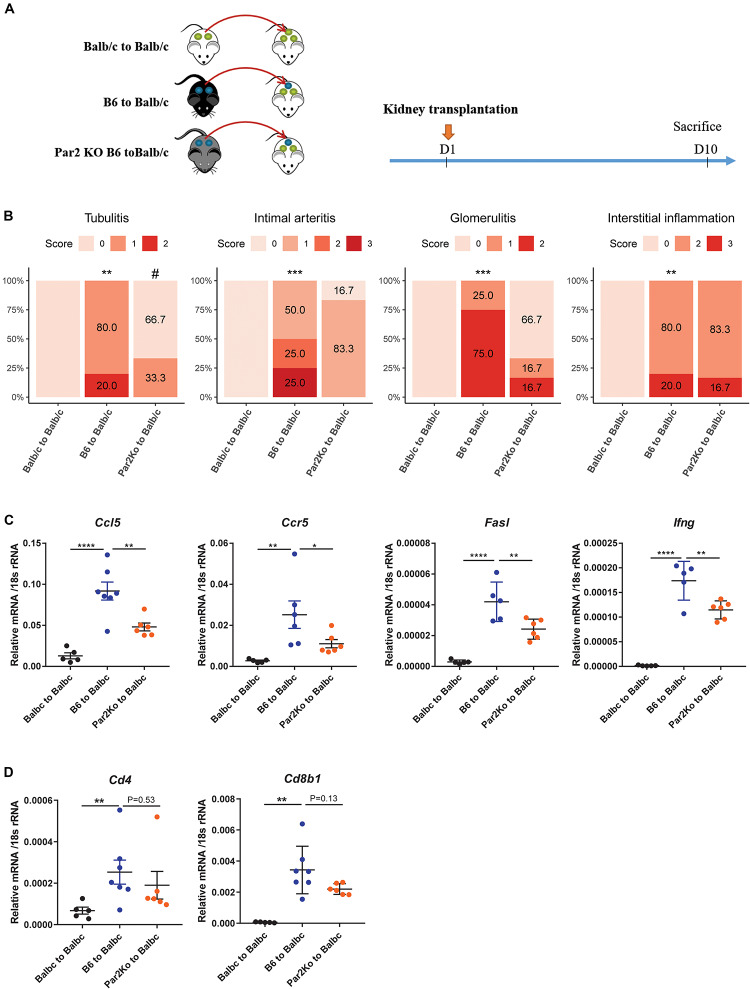
*Par2* deficiency in grafts attenuated renal allograft rejection *in vivo*. **(A)** Experimental design. Wild-type Balb/c kidneys (syngeneic) or wild-type C57BL/6 (allogeneic) were transplanted to wild-type Balb/c mice. *Par2*-deficient kidneys from C67BL/6 background (allogeneic) were transplanted into wild-type Balb/c mice. At day 10 after transplantation, mice were sacrificed for analysis. **(B)** Histological score for mouse kidney grafts. Tubulitis, intimal arteritis, glomerulitis, and interstitial inflammation were quantified by the Banff scoring method. **p < 0.01 B6 to Balb/c vs. Balb/c to Balb/c, ***p < 0.001 B6 to Balb/c vs. Balb/c to Balb/c, ^#^*p* < 0.05 B6 to Balb/c vs. Par2KO to Balb/c. *N* = 4 for Balb/c to Balb/c, *n* = 4–6 for B6 to Balb/c, *n* = 6 for Par2KO to Balb/c. **(C)** Proinflammation gene expression in mouse kidney grafts by RT-qPCR. **(D)** *Cd4* and *Cd8b1* gene expression in mouse kidney grafts. *p < 0.05, **p < 0.01, ***p < 0.001, ****p < 0.0001. B6 represents for C56BL/6J. Par2KO represent for *Par2* deficiency.

PAR-2 is a G protein receptor and acts as the receptor for many extracellular enzymes, such as Cat-S, trypsin, and tryptase. The Cat-S/PAR-2 axis was previously reported to play a role in itch, pain, and diabetic microvasculopathy (Kumar et al., 2016; Zhao et al., 2014; Mihara et al., 2016). Interestingly, using single-cell sequencing data of human kidney allograft rejection, we found F2RL1, the human gene encoding for PAR-2 to be expressed at low levels by several renal parenchymal cell types including endothelial cells and tubular epithelial cells (Figures 1B lower, 1C right). We therefore asked whether PAR-2 also plays a role in this setting. We designed the animal experiment as shown in Figure 5A. Compared to kidneys from wild-type B6 donors, kidneys from *Par2*-deficient donor mice showed significantly less tubulitis, non-significant trends toward less glomerulitis, and less intimal arteritis, and no effect on interstitial inflammation (Figure 5B). However, *Par2*-deficient allografts also showed reduced expression of the inflammatory genes *Ccl5*, *Ccr5*, *Fasl*, and *Ifng* (Figure 5C). In contrast to mice treated with Cat-S inhibitor, Par2-deficient allografts did not show reduced mRNA expression of Cd4 and Cd8b1 (Figure 5D) and of *Foxp3* and Il-2ra (Supplementary Figure S1C). Together, donor *Par2* deficiency attenuates acute allograft rejection.

## Discussion

We had hypothesized that interfering with either Cat-S or PAR-2 would attenuate kidney injury in a robust model of renal allograft rejection. We tested this concept using a pharmacological inhibitor of Cat-S and genetic *Par2* deficiency in different versions of the same kidney transplant model in mice based on the two strains C57Bl/6J (H2b) and Balb/c (H2d). By avoiding additional immunosuppressive therapy, we tested the role of these targets in severe acute rejection. The results confirm the hypothesis and identify Cat-S/PAR-2 as potential molecular targets in the context of solid organ transplantation.

Alloantigen recognition is a central path mechanism of alloimmunity. Both donor- and recipient-derived antigen-presenting cells activate recipient alloreactive T cells to proliferate and circulate, a process leading to alloantigen recognition inside the graft and local alloimmunity, i.e., rejection. These processes involve both MHC class I and II molecules of which peptide loading into MHC class II but not class I molecules is controlled by Cat-S (Rise et al., 1996). In support of this concept, Cat-S inhibition suppressed lymphocyte proliferation in a mixed lymphocyte assay, an accepted *in vitro* model of alloantigen recognition (Ansari and Strom, 2010). Our *in vivo* data further demonstrate that preemptive Cat-S inhibition was sufficient to suppress some aspects of acute renal allograft rejection, namely, tubulitis and arteritis, while, e.g., interstitial inflammation was hardly affected. This may relate to the contribution of MHC class I–mediated alloantigen recognition, and this suggests that monotherapy with a Cat-S inhibitor may be insufficient to control allograft rejection. In this regard, alloimmunity significantly differs from autoimmunity, which can be well controlled by Cat-S inhibitor monotherapy (Rupanagudi et al., 2015; Tato et al., 2017).

However, we and others have previously shown that Cat-S inhibition also prevents Cat-S-mediated activation of PAR-2 on vascular endothelial cells and thereby attenuates endothelial dysfunction–related organ injury (Kumar et al., 2016). In the setting of kidney transplantation, this would imply a potential dual renoprotective effect of Cat-S inhibition, one on alloantigen recognition and one of microvascular injury in the allograft. Indeed, our data show a considerable renoprotective effect of genetic *Par2* deficiency in the allograft. This genetic approach overcomes some of the concerns related to small molecule inhibitors such as exposure, dosing, dosing intervals, and specificity. Nevertheless, we found similar renoprotective effects as compared to the Cat-S inhibitor, indicating that the renoprotective effects of the Cat-S inhibitor largely relate to its activity at PAR-2. However, *Par2* deficiency also abrogates the activity of other serum proteases such as thrombin, which can have similar biological effects (Mihara et al., 2016). Although specific targeting of PAR-2 with drugs would be feasible, in the setting of solid organ transplantation targeting the dual activity of Cat-S appears more promising, potentially in combination with an immunosuppressive drug that also controls MHC class I–mediated alloimmunity.

Obviously, our study presents with some limitations. First, we could not ultimately prove that the *Par2*-dependent effects relate to Cat-S activity. Also, other proteases such as thrombin induce PAR-2 signaling on endothelium and tubular epithelial cells (Vesey et al., 2013; Mihara et al., 2016). Second, because of the robust nature of the acute rejection, it was difficult to quantify immune cell infiltrates and endothelial integrity properly and the life-non-sustaining transplantation technique did not allow testing for renal function. Finally, it would have been desirable to validate the experiments with Cats-deficient mice but such mice fulfilling the microbial requirements of our animal facility could not be obtained.

Together, Cat-S and PAR-2 are potential molecular targets in acute renal allograft rejection. Further studies will evaluate its potential in models that mimic more closely the clinical scenario of acute (and chronic) human allograft rejection.

## Data Availability Statement

The datasets generated for this study can be found in the GSE109564 and GSE114156.

## Ethics Statement

The studies involving human participants were reviewed and approved by Committee board University of Geneva (CEREH number 03-081). The patients/participants provided their written informed consent to participate in this study. The animal study was reviewed and approved by Regierung von Oberbayern, Germany.

## Author Contributions

JA, SK, and H-JA designed the study. BE, SK, and YL performed the mouse study. SuM performed the Banff scoring of mouse kidney. YL and SK performed the *in vitro* study and the mouse sample analysis except Banff scoring. SoM performed the immunostaining of human biopsies. AM and BH performed the single-cell sequencing. YL and H-JA prepared the manuscript. All authors revised and approved the final version of the manuscript.

## Conflict of Interest

The authors declare that the research was conducted in the absence of any commercial or financial relationships that could be construed as a potential conflict of interest.

## Supplementary Material

FIGURE S1 **(A)** Gating strategy for proliferation analysis of mixed lymphocyte reaction. **(B)** Foxp3 and Il2ra gene expression in mouse kidney grafts from mice treated with Cat-S inhibitor or vehicle. **(C)** Foxp3 and Il2ra gene expression in mouse kidney grafts from wild type or Par2 deficient mice. **(D)** Representative images of MHC-II staining in mouse kidney grafts treated with Cat-S inhibitor or vehicle. Magnification, 200×.
