# Supplementary figures and images for: Cathepsin S and Protease-Activated Receptor-2 Drive Alloimmunity and Immune Regulation in Kidney Allograft Rejection
Source: Front Cell Dev Biol. 2020 Jun 5;8:398. doi: 10.3389/fcell.2020.00398 (PMC7290053; doi:10.3389/fcell.2020.00398)

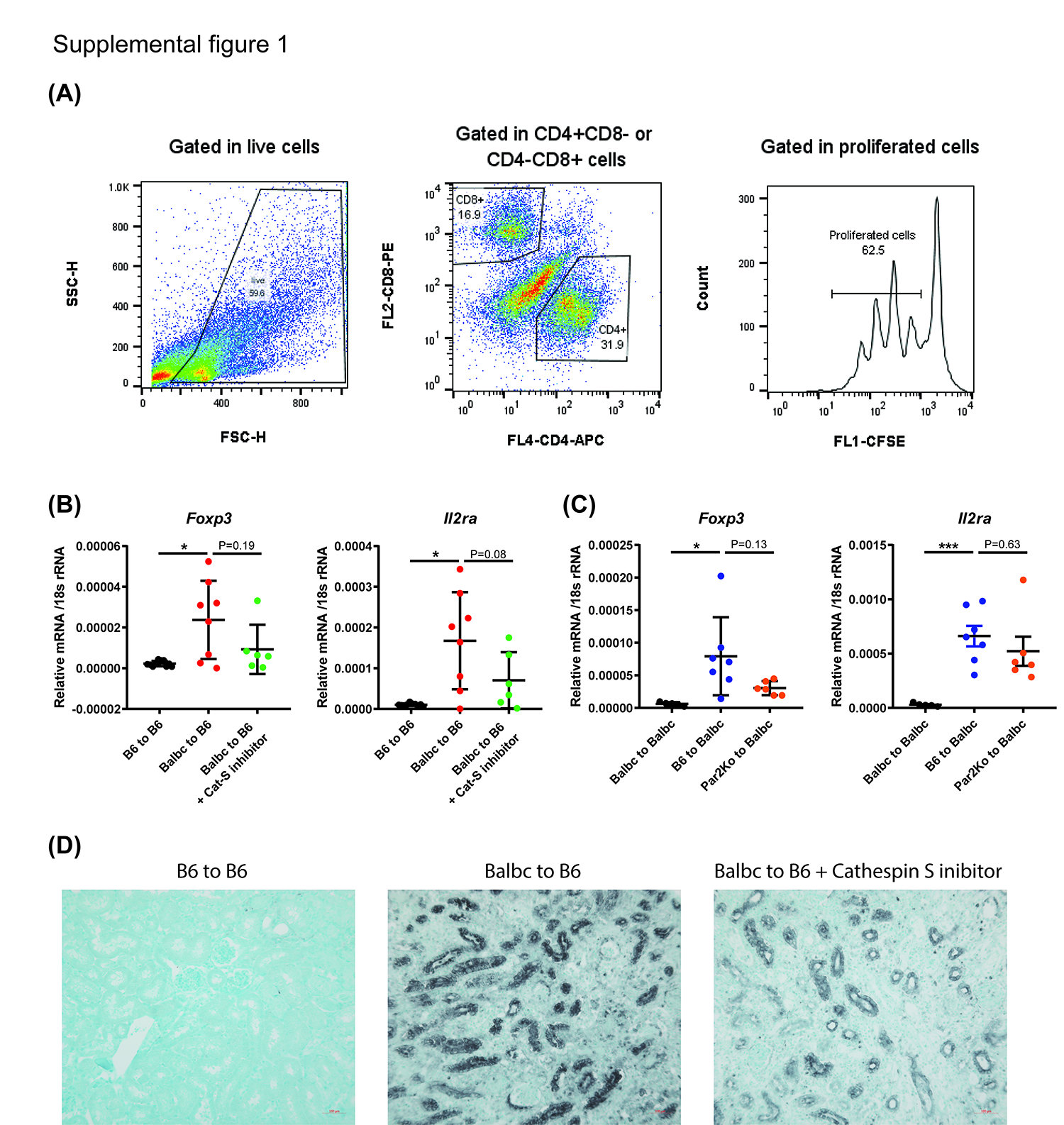

Supplement: FIGURE S1 — (A) Gating strategy for proliferation analysis of mixed lymphocyte reaction. (B) Foxp3 and Il2ra gene expression in mouse kidney grafts from mice treated with Cat-S inhibitor or vehicle. (C) Foxp3 and Il2ra gene expression in mouse kidney grafts from wild type or Par2 deficient mice. (D) Representative images of MHC-II staining in mouse kidney grafts treated with Cat-S inhibitor or vehicle. Magnification, 200×. [file Image_1.TIF]
